# A modified rat model of hindlimb ischemia for augmentation and functional measurement of arteriogenesis

**DOI:** 10.14440/jbm.2018.234

**Published:** 2018-04-10

**Authors:** Ryan M. McEnaney, Dylan McCreary, Edith Tzeng

**Affiliations:** 1 Department of Surgery, University of Pittsburgh School of Medicine, Pittsburgh, PA 15260, USA; 2 Veterans Affairs Pittsburgh Healthcare System, Pittsburgh, PA 15240, USA

**Keywords:** arteriogenesis, collateral, hindlimb ischemia, imaging

## Abstract

Arteriogenesis (collateral artery development) is an adaptive pathway critical for salvage of tissue in the setting of arterial occlusion. Rodent models of arteriogenesis typically involve an experimental occlusion (ligation) of a hindlimb artery and then rely on indirect measures such as laser Doppler perfusion imaging to assess blood flow recovery. Unfortunately, the more commonly utilized measures of distal tissue perfusion at rest are unable to account for hemodynamic and vasoactive variables and thus provide an incomplete assessment of collateral network capacity. We provide a detailed description of modifications to the commonly used model of femoral artery ligation. These serve to alter and then directly assess collateral network’s hemodynamic capacity. By incorporating an arteriovenous fistula distal to the arterial ligation, arterial growth is maximized. Hindlimb perfusion may be isolated to measure minimum resistance of flow around the arterial occlusion, which provides a direct measure of collateral network capacity. Our results reinforce that arteriogenesis is driven by hemodynamic variables, and it can be reliably augmented and measured in absolute terms. Using these modifications to a widely used model, functional arteriogenesis may be more directly studied.

## INTRODUCTION

Arteriogenesis is the natural process of collateral artery development which occurs in response to flow limiting arterial occlusive disease. In humans, arteriogenesis is life and limb-saving, as the diversion of blood flow can maintain tissue perfusion in the face of major arterial occlusions. Without collateral arteries, individuals challenged with such occlusions would suffer profound ischemia of brain, myocardium, skeletal muscle and abdominal viscera. Despite the importance of spontaneously formed collateral arteries, they fail to completely restore the blood flow capacity that is lost with large artery occlusion [1,2]. An explanation is that the driving force of arterial remodeling into collateral arteries is shear stress, a weak driving force that is rapidly extinguished as the vessel enlarges.

Functional collateral arteries are known to develop from preexisting inter-arteriolar connections between adjacent parent arteries, the extent of which is genetically determined [3,4]. Interestingly, arteriogenesis has also been demonstrated to have potential for increasing collateral network capacity beyond what is spontaneously achieved, although these studies have required use of large animal models [5,6]. If therapies to stimulate enhanced arteriogenesis (beyond the spontaneous collateralization) could be identified, a much needed medical alternative to surgical revascularization for patients suffering from arterial insufficiency may be found.

Rodent femoral artery ligation (FAL) is a widely used surgical model to evaluate arteriogenesis. Typically, the common femoral artery and some of the distal arterial tree of the hindlimb is exposed and ligated or excised, perturbing flow patterns and stimulating dilation and remodeling among arteriolar interconnections bridging territories. Laser Doppler perfusion imaging (LDPI) is an effective and established method to assess target tissue perfusion recovery, but does not provide collateral vessel morphology [7]. Collateral vessels themselves may be imaged conventionally through tissue sectioning and immunohistochemistry, whole-tissue imaging techniques such as arteriography or contrast-enhanced micro-computed tomography (micro-CT) or *in vivo* imaging technology such as optical coherence tomography-based microangiography [8-11]. Magnetic resonance imaging techniques have been used to quantitatively assess structure and flow of collateral networks in living animal models of peripheral arteriogenesis [12-14]. Even these sophisticated imaging methods have limitations accounting for numerous hemodynamic, anatomic and vasoactive variables that may obscure the true capacity of the developed collateral network. The anatomic variation between animals, which occurs in higher frequency among smaller branch vessels and arterioles, can make interpretation of morphometric data problematic.

In this report, we provide detailed description of modifications to an established FAL technique in a rat model. First, we construct an arteriovenous fistula beyond the arterial ligation, thereby increasing shear stress applied to the collaterals. We show that this results in enhanced collateral artery remodeling and capacity compared to ligation alone. Next, we describe a technique to simultaneously transduce arterial pressures on both sides of the arterial occlusion, permitting calculation of functional resistance for the entire collateral network. This method is analogous to the pressure-derived collateral flow index at the time of coronary interventions, a prognostically useful measure of coronary collateral function in humans [15]. We demonstrate that use of these techniques result in a collateral network which achieves a greater functional capacity, and at a faster rate, than the collaterals that form from FAL alone. This platform would be useful for evaluating potential therapies designed to improve arteriogenesis as a strategy for revascularization of an ischemic organ.

## MATERIALS AND METHODS

### Femoral arterial ligation with arteriovenous fistula

Male adolescent Sprague-Dawley rats approximately 300–350 g were purchased from Harlan Laboratories (Indianapolis, IN), age matched and raised under pathogen-free conditions. All animal procedures were performed in accordance with the Institutional Animal Care and Use Committee of the University of Pittsburgh (Protocol #17030207). All animals were housed in the animal facilities at the University of Pittsburgh.

Rats were anesthetized with intraperitoneal ketamine (80 mg/kg) and xylazine (10 mg/kg). Adequacy of anesthesia was determined by respiratory rate and reaction to toe pinch. Supplemental inhalational isoflurane was delivered by nosecone as necessary. Abdominal and inguinal hair was removed with clippers and depilatory cream. The skin was prepared with betadine solution and sterilely draped for the survival procedures. Post-operatively, animals were given 0.1 mg/kg buprenorphine subcutaneously for analgesia every 12 h thereafter for a total of 48 h.

### Femoral artery ligation with arteriovenous fistula

A longitudinal inguinal skin incision is made directly overlying the femoral vessels. The femoral fat pad is first divided and the superficial epigastric artery and vein are ligated with 6-0 silk ligatures and divided to facilitate exposure of the common femoral vessels. The common femoral artery is then exposed from the inguinal ligament to the femoral bifurcation with using fine forceps and tissue scissors. Exposure is facilitated by the use of a small retractor to retract the inguinal ligament and the abdominal wall medially. Occasionally, it is necessary to divide the inguinal ligament to improve exposure, taking care not to injure the inferior epigastric vessels just deep to the abdominal wall. The common femoral artery and vein are skeletonized from the inguinal ligament to their bifurcations. This may be done bluntly with fine forceps. A segment of common femoral vessels without intervening branches is isolated. (Note that a circumflex femoral artery with accompanying vein is typically present in the proximal common femoral artery.) This segment of common femoral artery and vein are then double-encircled with 6-0 silk suture without tying, and small plastic clips or fine hemostats are applied to keep the strands together.

**Figure 1.**
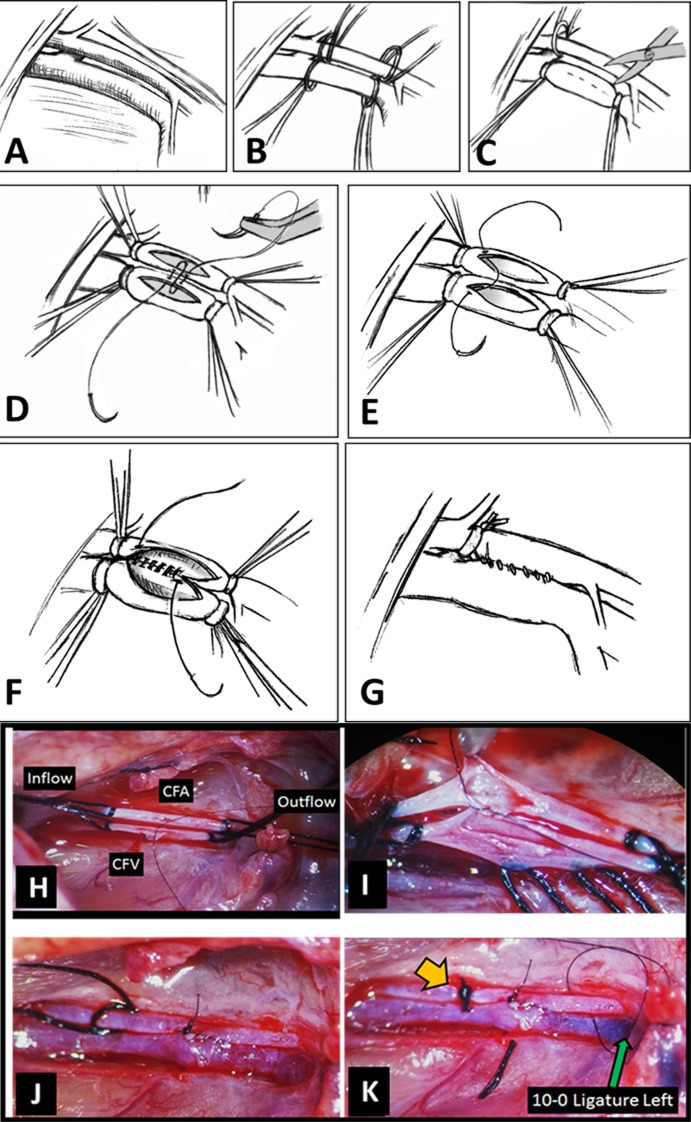
Illustration of femoral ligation with distal arteriovenous anastomosis. **A** and **H.** Common femoral artery and vein are exposed, the femoral nerve is located cephalad to the artery. **B.** The common femoral artery and vein are isolated and controlled with a double-looped suture such as 6-0 silk. **C.** The femoral vein is occluded by tightening the 6-0 loops and then opened with a micro-scissors to create a linear venotomy. A catheter may be easily inserted for administration of heparinized solution. An adjacent linear arteriotomy may be created in a similar fashion. **D** and **I.** Using a double-armed, fine monofilament suture (9-0 or 10-0), the anastomosis is begun on the back wall and run outward in each direction. **E.** Alternatively, a single arm suture may be used if the anastomosis is begun at one corner in the near wall and then run along the back wall to the opposite corner (F) before returning. **G** and **J.** The anastomosis is completed with and the suture tied with squared knots. The proximal femoral artery loop is left in place, and the remaining 6-0 silk ligatures are removed. Note the lightened arterialized flow in the vein demonstrating fistula patency in (J) and (K). The proximal femoral artery is then ligated with the 6-0 silk (yellow arrow). Flow from the distal artery then proceeds through the fistula. Mixed blood is seen in the proximal vein (appears pink) versus the distal vein (blue) in image (K). A loop of 10-0 monofilament may be left in place to facilitate closure of the fistula at a later time point (green arrow).

The femoral vein is temporarily occluded by tightening the silk loops and a linear venotomy is created using micro-scissors. The vein is then cannulated with a micro-renathane catheter of 0.01–0.025” diameter tubing attached to a blunt-tipped needle (Braintree Scientific, Braintree, MA). Cutting the catheter tip at an angle can aid in placement of the catheter in the lumen. It is important to flush all tubing of air before cannulation. Heparin (100–150 units in 0.9% NaCl) is administered slowly through the femoral vein catheter for systemic anticoagulation, taking care not to introduce air into the circulation. Once heparinized, the femoral artery is then also temporarily occluded by tightening the silk loops. Care must be taken to ensure that the vessels are occluded, without undue tension. Typically, placing a lightweight hemostat clamp on the end of the silk thread and allowing it to hang freely outside of the wound is adequate. Occasionally the loop will loosen and need to be retightened to maintain arterial control. A linear arteriotomy is made in the common femoral artery opposite to the femoral venotomy in with micro-scissors. Using a 9-0 or 10-0 double-armed monofilament nylon suture (Ethicon, Somerville, NJ), an arteriovenous anastomosis is created in a side-to-side manner (**Fig. 1**). This is most easily accomplished by beginning with an inside-to-out placement in the back wall of the artery and vein, and then proceeding around the corners to the front wall to complete the anastomosis. The suture is then secured by tying the strands together with forceps (**Fig. 1**). It is preferable to orient the needle placement so that the inside-out needle passes occur in the distal femoral artery and proximal femoral vein. Alternatively, a single-armed 9-0 suture (ARO Surgical, Newport Beach, CA) may be used when the anastomosis is started along the front wall and run across the back wall first. The anastomosis may be partially completed with the femoral vein catheter in place, which will ensure maintenance of adequate flow lumen while sewing. After completing the anastomosis, suture is tightened and instrument tied using fine forceps and divided. Flow is then reestablished from the femoral artery and vein. Patency of the fistula can be identified immediately as mixing of arterial and venous blood is easily visible in the outflow vein (**Fig. 1J**). After assuring hemostasis with gentle compression using a cotton-tipped applicator, the proximal common femoral artery is ligated with 6-0 silk suture. The final result is a femoral artery ligation with a distal arteriovenous fistula (**Fig. 1G** and **1K**). The wound is irrigated with saline and closed in 2 layers using: (1) a monofilament suture for the deep subcutaneous tissue, and then (2) an interrupted horizontal mattress closure of the skin using monofilament suture. Animals are awakened from anesthesia and recovered on a warming pad. Following this, animals were observed frequently to monitor function as well as follow for signs of distress. Animals demonstrating signs of distress were euthanized following Institutional Animal Care and Use Committee (IACUC) protocols.

**Figure 2.**
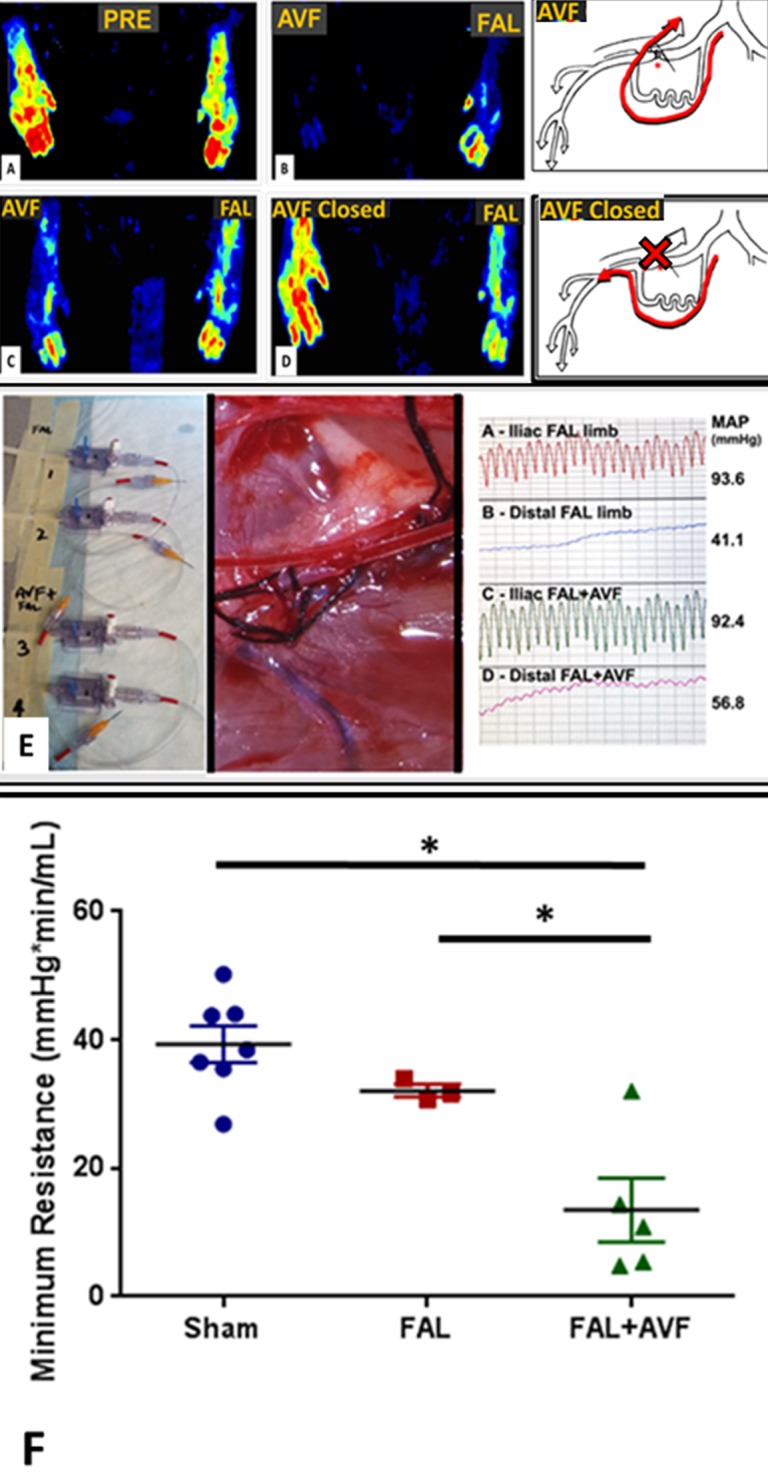
Arteriovenous fistula augmentation of arteriogenesis. **A-D.** Laser Doppler perfusion images demonstrate AVF function. Perfusion heatmap immediately before (A) and after (B) surgical FAL versus FAL + AVF. After 2 weeks, there is considerable recovery in the heatmap perfusion for both lower limbs (C). However, hindlimb recovery appears relatively better in the FAL limb, and worse with the AVF, due to ‘steal’ from the functional AVF. After surgical closure of the fistula, perfusion through the better developed collaterals is demonstrated (D). Image of parallel pressure transducers and image of microcatheterization of the proximal and distal femoral artery. **E.** Pressure transductions after closure of the fistula show a significant increase in mean arterial pressure and pulsatility transmitted by the enhanced collateral network. **F.** Minimum resistance is calculated for the collateral network of hindlimbs. At 7 d, FAL + AVF limbs demonstrate a significant reduction in minimum resistance across this collateral system, with respect to FAL alone. (*P* = 0.0007, one way ANOVA).

After a period of recovery ranging between 1–2 weeks, the animals are anesthetized again as above. The prior incision is opened and the femoral vessels again identified and dissected free from surrounding tissue using blunt dissection with forceps and a cotton-tipped applicator and micro-scissors. Care must be taken with repeat exposure because scar tissue and wound hyper vascularity may increase risk of bleeding. The distal femoral artery, beyond the arteriovenous anastomosis, must be identified and encircled with 6-0 or finer ligature. Ligation of the artery in this location effectively closes the fistula, such that all arterial flow reentering the femoral artery *via* collaterals may perfuse the paw (**Fig. 2**).

### Laser Doppler perfusion imaging

At time points of 7, 14, and 21 d, animals are reassessed and are anesthetized as described above. LDPI is obtained on the anesthetized animals using the PeriScan III instrument (PeriMed, North Royalton, OH). Rats are placed on a warming pad for 5 min. Six consecutive images are obtained of the hind limb and paw by LDPI. After initial imaging, the femoral vessels are then re-explored and the arteriovenous fistula (AVF) is ligated as described. This redirects all collateral network flow into the hindlimb. Repeat LDPI is then performed after the animal has been allowed to acclimate to the warming pad for another 5 min. Six additional images were obtained. The images pre-and post-fistula ligation are then compared. Region of interest is defined as the paw and perfusion intensity measurements are compared.

### Hemodynamic measurement of collateral networks

The individual arterial pressure transducers (TruWave, Edwards LifeSciences, Irvine, CA) are calibrated and established in parallel using a PowerLab 4/30 and LabChart software (AD Instruments, Colorado Springs, CO). The rat is anesthetized and prepped as described previously. The femoral vessels are completely exposed for both hindlimbs and double-encircled with separate 6-0 silk sutures. The 6-0 silk loops are used to temporarily occlude the proximal and distal femoral artery. A femoral arteriotomy is then created using microscissors. The 0.025'' diameter renathane catheters (Braintree Scientific) are inserted into the proximal and distal femoral arteries, and are flushed with heparinized saline, ensuring that no air is trapped within the tubing. The rat is slowly given 100–150 units of intravenous heparin given through the proximal arterial catheter. Catheters are secured in place by tightening the 6-0 silk loops and are then attached to fluid-locked pressure tubing connected to a pressure transducing sensor. Simultaneous pressure waves are transduced from proximal and distal artery catheters in each hindlimb. After intravital pressures and tracings are recorded (**Fig. 2E**), the animal is euthanized through exsanguination, venting the right atrium. The abdominal aorta is dissected *via* midline laparotomy and encircled with 6-0 silk ligature. An aortotomy is made with microscissors, and the aorta below the renal arteries is catheterized with a 0.037'' renathane microcatheter and secured with a silk suture. Blood volume is flushed with heparin saline. Finally, a vasodilator perfusate (comprised of PVP40 (58.8 g/L), 100 μM adenosine, 100 μM sodium nitroprusside, and 100 μM verapamil) warmed to 37°C is administered into the aorta. Using a programmable syringe pump, the perfusate is administered at set flow rates of 3, 5 and 7 cc per minute. Intra-arterial pressures are recorded both proximal (P1) and distal (P2) to the occlusion under each flow rate (*Q*). Resistance of the collateral circuit (*Rcol*) can be calculated using Ohm’s law:

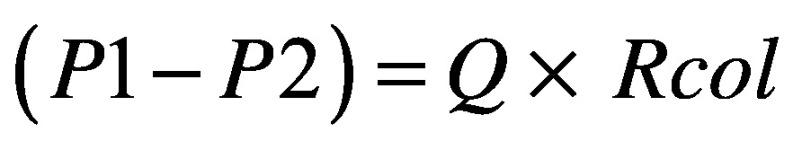



*Rcol* can be solved for: 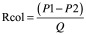


Where *Rcol* is expressed in resistance units, or 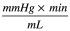.

### Microfil casting

Pigmented vascular casts are created using Microfil (MV120-blue; Flow Tech Inc., Carver, Mass) infused into the abdominal aorta following euthanasia. Microfil is prepared according to manufacturer’s instructions. The aorta is flushed with heparinized saline containing adenosine, verapamil and nitroprusside to remove blood elements and to maximally vasodilate the vasculature. Microfil is then infused under direct visualization until free flow through the femoral veins is observed. The IVC and aorta are then ligated to prevent Microfil escape from the vasculature, and the specimen was allowed to cure at 4°C for 24 h. Hind limb dissection of the cured specimens is conducted to expose superior thigh and pelvic/adductor collateral stems. Images are obtained under magnification using an operating microscope fitted with a calibrated optical micrometer. Collateral artery diameter is measured and compared between FAL, AVF + FAL, and sham operated arterioles.

### Post-mortem CT arteriogram

Specimens were prepared similarly to Microfil casting as above. Fixed, casted specimens were imaged with a multi-modal Siemens Inveon micro-CT system (Inveon, Siemens Inc., Knoxville TN, USA) with parameters optimized for image acquisition at 39 µm pixel size, 400 µA, 800 ms exposure. From raw images, volume rendering images were reconstructed for further evaluation and diameter measurements.

### Statistical analysis

Results are expressed as mean ± standard error of the mean. Comparisons are performed using an independent *t*-test or one way ANOVA. *P* values < 0.05 are considered statistically significant. All calculations were performed in GraphPad statistical software suite.

## RESULTS

### Functional and perfusion recovery

After FAL, all animals were followed for walking behavior, and all animals recovered functional use of the ischemic hindlimbs within 2–3 d. No animals developed severe cutaneous tissue ischemia or gangrene. In total, three LDPI data points were obtained: prior to surgery, immediately after, and at seven days of recovery. Immediately after FAL, there is an expected, severe drop in tissue perfusion of the paw. This drop is exacerbated by the creation of an AVF distal to the ligation (**Fig. 2A** and **2B**). By day 7, hindlimb perfusion had recovered significantly in both hindlimbs. However, LDPI consistently demonstrated that the limb with both a FAL and a patent AVF have much less perfusion to the foot compared with the hindlimb recovering from FAL only (**Fig. 2C**). This finding is due to the patent fistula “stealing” perfusion from the hindlimb, as the resistance of the venous system is far less than that of the capillary network. The collateral network is actually capable of a much greater conductance of flow. Once selective ligation of the AVF is performed, perfusion to the paw is significantly greater on the limb with the “trained” collateral vessels (**Fig. 2C-2D**). Rats receiving a femoral AVF in addition to FAL exhibited a 24.2% increase in perfusion to the hind limb after 2 weeks of recovery when compared to the FAL only limb (*P* = 0.006, two-tailed *t*-test).

### Collateral resistance and conductance

Successful catheterization and pressure transduction was established for all animals. The mean arterial pressure of all rats after ligation of both hindlimbs was 130.4 ± 5.6 mmHg. Transduction of arterial pressures in the live animal across these networks provides further evidence that the “trained” collaterals remodeled to a greater capacity than the “untrained” vessels. In **Figure 2E**, arterial pressure and pulsatility are demonstrated. There is a significant increase in both distal pressure and pulsatility in the distal femoral artery of the fistula “trained” limb when compared to the “untrained” limb (**Fig. 2B** *vs*. **Fig. 2D**). Notably, there was significant variation of the distal arterial pressures of animals under anesthesia. Minimum resistance was then measured for the collateral circuit of each limb. After one week of recovery, there is a modest but significant decrease in mean minimum resistance of the collateral network after FAL compared to sham operation (32.1 ± 1.0 *vs*. 39.3 ± 2.8 mmHg/ml*min, *P* = 0.046). The minimum resistance was greatly reduced, by comparison, among collateral networks exposed to FAL + AVF treatment (13.7 ± 5.1 mmHg/ml*min, *P* < 0.02) (**Fig. 2**). Calculation of maximum collateral conductance at the measured mean arterial pressure yielded 3.32 ml/min for sham operated limbs, 4.06 ml/min for FAL limbs, and 9.52 ml/min for FAL + AVF limbs (at 130.4 mmHg).

### Collateral size

**Figure 3.**
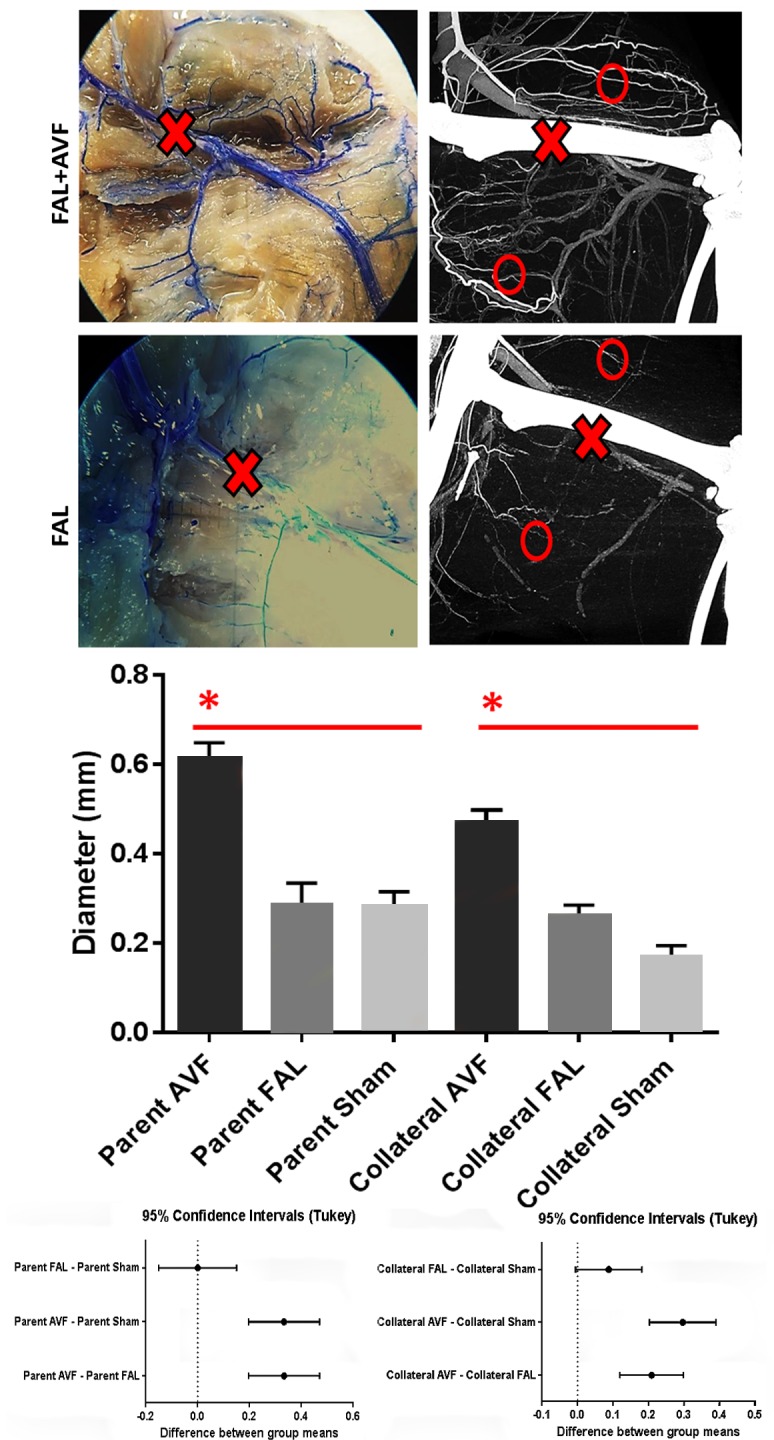
Morphometric analysis of collateral vessels. Microfil vascular casting was performed and imaged *via* surgical microscopy (left) and micro CT (right). Larger and more numerous collateral vessels are demonstrated after treatment with FAL + AVF versus FAL alone. Location of arterial ligation denoted with “x”. Examples of third order collateral branches marked with red circle. Graph demonstrates the > 100% increase in parent artery and collateral vessel diameter with respect to sham or FAL alone. *Significant with *P* < 0.0001 by one way ANOVA.

Collateral artery diameter was measured in rats undergoing FAL, AVF + FAL, and sham operation. Parent arteries for major collateral artery groups observed include the internal iliac artery and reconstitute the deep femoral artery or the iliacofemoral artery to reconstitute the saphenous and popliteal arteries. CT scans demonstrate qualitatively increased number of visible vascular structures across the thigh of FAL specimens with respect to Sham operated hindlimbs, to recapitulate distal perfusion (**Fig. 3**). Among AVF + FAL hindlimbs, this is further increased. Collateral arteries were analyzed for number using maximal intensity projections and multiplanar reconstructions to measure vessel diameter. Among parent arteries such as the proximal caudal femoral artery or saphenous artery, there was a greater than two-fold increase in diameter when compared to FAL alone or sham-operated limbs, although there was no significant difference between FAL versus sham. Within the collateral arteries (≥ 3 branch points removed from the parent artery) there was a similar size increase in mean diameter of the AVF enhanced limb versus the FAL limb. There is a non-statistically significant difference between the FAL and sham limbs by one-way ANOVA. Confidence intervals are plotted and are approaching significance for the FAL versus sham comparison.

## DISCUSSION

Collateral network enhancement by therapy targeting arteriogenesis represents a promising pathway to nonsurgical revascularization for patients with arterial occlusive disease. To better understand arteriogenesis, we must be able to study the early events relating to flow and shear stress and how they translate to outward vascular remodeling of developing collateral vessels. Unfortunately, frequently used methods for studying arteriogenesis in small animal models are unable to measure collateral network capacity, and large animal models may be prohibitively expensive [6]. We have described an accessible rodent model of arteriogenesis where the functional capacity of collaterals may be greatly enhanced through AVF placement, and measured directly in a precise and highly reproducible way.

Established methods for analyzing arteriogenesis in rodent models often involve measurement of perfusion recovery over time by LDPI. LDPI is based upon the principle that a “Doppler shift” occurs when laser light is scattered from a moving object, namely, a red blood cell (RBC) [16]. The average frequency of the Doppler shift fluctuation is proportional to the average velocity of the RBCs, whereas the amplitude of the fluctuation is proportional to the number of RBCs scattering light. Blood flow is computed as the product of the mean velocity and number of moving blood cells and is expressed in arbitrary perfusion units mapped to an image. Tissue penetration is limited and dependent on the incident laser wavelength, with the typical visible red light (~680 nm) capable of approximately 1 mm of tissue penetration, thus limiting measurements to capillary level flow of the dermal papillae. As such, perfusion recovery measured by LDPI cannot distinguish true collateral artery growth (arteriogenesis) from increased tissue capillary density (angiogenesis). Additionally, LDPI instruments cannot be calibrated to absolute units of blood flow. To counter, measurements are typically reported as ratios of perfusion of the recovering ischemic hindlimb to a sham limb as a control. This practice allows vasoactive variables (which act differently on the collaterals of the ischemic hindlimb versus the arterioles of the control limb) to influence perfusion measurements. Extremity arterial flow is typically high resistance in the resting state, the point at which LDPI is performed. Warm hyperemia is often used to limit the peripheral vasoconstriction, as cutaneous perfusion measurements are made beyond the distal arteriolar beds. Strategies to comprehensively analyze the collateral network in its entirety, taking into account the size and shape of all relevant collateral pathways, are an alternative [8]. One disadvantage is that the image data are cumbersome to analyze, limited by the image acquisition modality and do not directly establish the functional collateral network capacity. Morphometric analysis alone may be further confounded as vessel diameters are dynamic and are subject to changes during live imaging and even postmortem processing.

To understand the underlying mechanisms of arteriogenesis, quantitative assessments which take into account the entirety of the collateral network may be helpful. As hemodynamic forces drive flow across numerous arteriolar interconnections, slight variations in geometry and anatomy between individual animals can lead to variations in dominant collateral artery development. In human coronary disease, pressure-derived collateral flow indices have been demonstrated to be of superior prognostic value to angiographic assessments of collateral circulation alone [15,17]. Likewise, hemodynamic measures of the collateral circuit such as those described here provide a functional assessment of collateral networks and accounts for normal anatomical variations in branching patterns and collateral patterning. In our study, we demonstrated that, while LDPI does measure improved perfusion in the limbs with FAL + AVF at 24% greater than FAL alone, hemodynamic measurements at maximal vasodilation revealed a 134% increase in maximum conductance. Therefore, the window of difference is much greater when comparing gains in conductance. In clinical translation, the total conductance of the collateral network should be of great importance, regardless of the vascular bed. In the coronary circulation, for instance, therapeutic arteriogenesis should be capable of increasing total collateral conductance to better meet the needs of increased myocardial demand under cardiac stress situations.

The technique described is a reproducible way to augment arteriogenesis and calculate total resistance (and thereby, conductance) for a collateral network using a rat model. These functional assessments provide measurements of collateral arterial networks that are clinically relevant and may be useful for studies of arteriogenesis. We also show that the augmentation of flow in a collateral network by creation of an AVF stimulates a reproducible and dramatic increase in its maximal conductance. Our findings support existing evidence that collateral development has a significantly higher ceiling beyond what is spontaneously achieved after arterial occlusion [5,6]. Consideration must be given to the limitations of these methods. It must be recognized that the arterial system is complex and cannot be completely isolated despite isolated perfusion through the iliac artery. Thus, during arterial perfusion, there is some loss to capillary transit and through arteriolar connections outside of the hindlimb, including splanchnic, cutaneous, and lumbar vascular beds.

Arteriogenesis is a complex process which depends upon cells of various tissues responding to mechanical stimuli [5,18-20]. Currently, no *in vitro* models of arteriogenesis have been developed. Even when modeled *in vivo*, arteriogenesis may be challenging to assess due to the complexity of the collateral networks that develop. As we have shown, collateral artery development has the capacity to achieve much greater growth with increased stimulation than spontaneously achieved. The methods described here provide a means to directly evaluate the functional relevance of collateral vessels while excluding off target effects, such as those affecting the microcirculation.
